# The gut virome and the relevance of temperate phages in human health

**DOI:** 10.3389/fcimb.2023.1241058

**Published:** 2023-07-27

**Authors:** Laura Avellaneda-Franco, Sofia Dahlman, Jeremy J. Barr

**Affiliations:** School of Biological Sciences, Monash University, Clayton, VIC, Australia

**Keywords:** gut temperate phages, gut prophages, VLPs metagenomes, bulk metagenomes, gut virome, phage bacteria interaction, phage bacteria mammalian cells interaction, culture-dependent techniques

## Abstract

Alterations in the gut virome impact human health. Bacteriophages, viruses that infect bacteria, dominate the gut virome and are mainly composed by virulent and temperate phages. While virulent phages exclusively replicate within and lyse their bacterial host’s cell, temperate phages switch from an integrated state residing within their bacterial host’s chromosome to an induced free virion state via an induction event. How often do these induction events occur and what are their implications on gut homeostasis? Here, we summarize the current knowledge of the gut virome based on metagenomics and present how the proportion of induced temperate phages varies amongst individuals, age, and disease states. Finally, we highlight the importance of building upon classical culture-dependent techniques and sequencing approaches to improve our understanding of temperate phages to enable their potential therapeutic use.

## Introduction

The human gut harbours a highly diverse and stable viral community that impacts human health (Shkoporov et al., 2019; Garmaeva et al., 2021; Mihindukulasuriya et al., 2021; Van Espen et al., 2021). These gut viruses predominantly consist of bacteriophages, which directly interact with both their bacterial hosts and the mammalian cells lining the gut (Jahn et al., 2019; Cornuault et al., 2020; Liang and Bushman, 2021), suggesting that viruses have a critical ecological role in the maintenance of gut homeostasis (Gregory et al., 2020; Li Y. et al., 2021). An increasing number of studies are investigating the association of the gut virome with several pathologies, including metabolic syndrome (de Jonge et al., 2022), non-alcoholic fatty liver disease (Lang et al., 2020), inflammatory bowel disease (IBD) (Clooney et al., 2019; Gogokhia et al., 2019; Ansari et al., 2020), diabetes (Zhao et al., 2017; Ma et al., 2018), colorectal cancer (Hannigan et al., 2018), and Parkinson’s disease (Tetz et al., 2018). Fluctuations in the gut viral community may represent novel biomarkers of disease states or therapeutic targets (Clooney et al., 2019; Mangalea et al., 2021). However, mechanistic links between these gut viruses and disease largely remain to be determined (Lang et al., 2020; Voorhees et al., 2020).

Coupled with next-generation sequencing, the enrichment of viral-like particles (VLPs) has provided a high-resolution lens with which we can study the highly diverse, unknown, and individual-specific viral community that constitutes the adult gut virome (Liang and Bushman, 2021). While more than 60% of gut viral contigs cannot be assigned to a taxonomic level, upwards of 97% of the contigs that can be assigned represent bacteriophages (Shkoporov et al., 2019; Garmaeva et al., 2021). Bacteriophages, or simply phages for short, are viruses that replicate within bacterial cells through a continuum of life cycles, with the lytic and lysogenic cycles being the most studied (Liang and Bushman, 2021). Within the lytic lifecycle, virulent phages lyse their bacterial host to release their viral progeny, whereas temperate phages have the potential to either undergo a lytic cycle or integrate into their bacterial host’s chromosome as a prophage, as part of the lysogenic lifecycle (Liang and Bushman, 2021).

**Figure 1:**
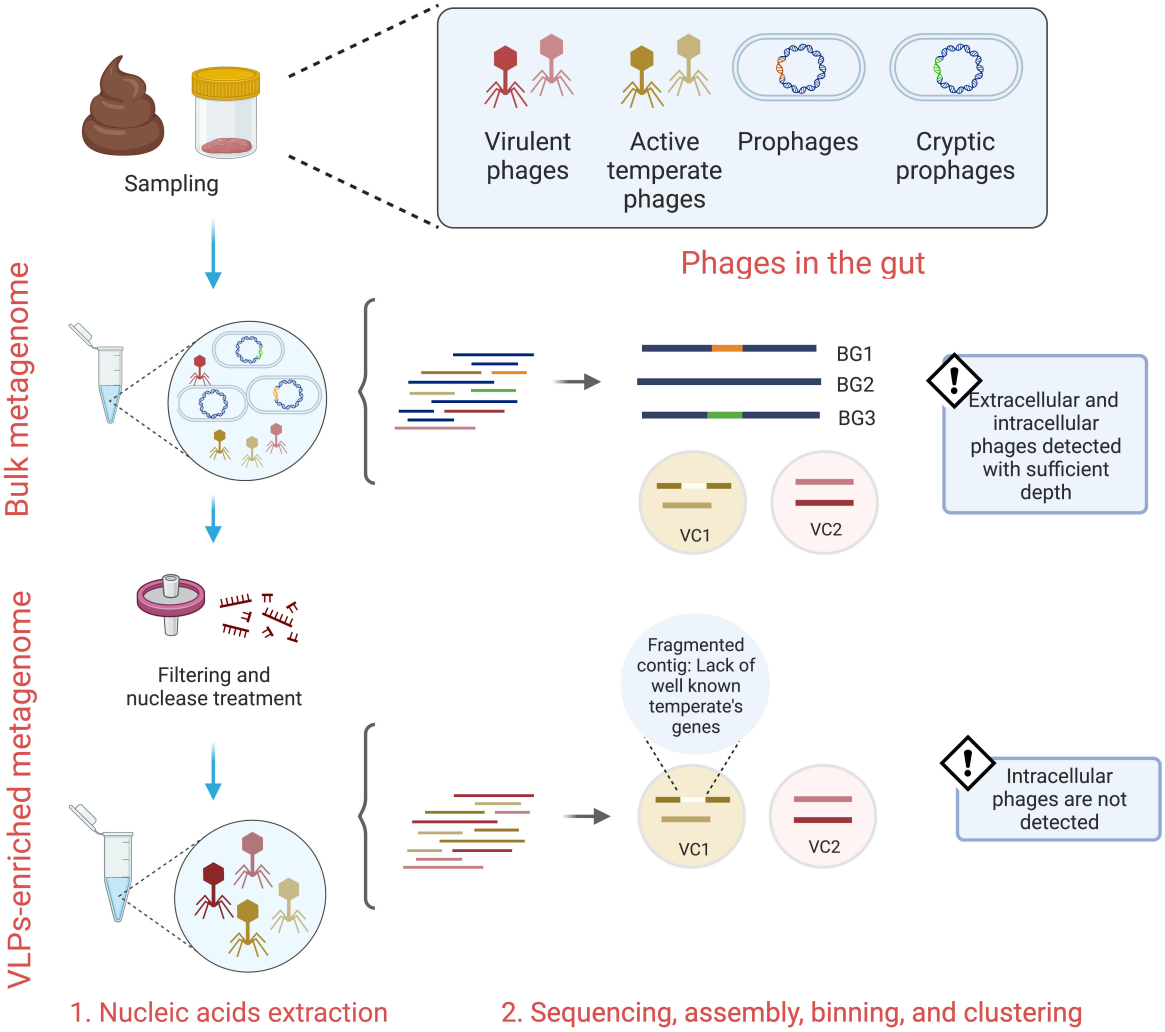
Bulk and VLPs-enriched metagenomes survey different gut phages lifestyles. While bulk metagenomes provide extracellular and intracellular phages sequences, VLPs-enriched metagenomes mostly provide sequences from extracellular phages. Both techniques depend on either homology-based methods or machine learning algorithms to predict phages and their lifestyles. The lack of extensive phage-encoded proteins databases, cryptic phages, and fragmented genomes lead to false positive results. BG, bacterial genome; VC, viral cluster.

When considering these phage lifecycles, several technical challenges emerge with the collection and subsequent analysis of lysogenic viruses from the gut. When processing gut VLPs, the captured virions comprise phages replicating only within the lytic stage of the phage lifecycle, thus integrated prophages are not directly surveyed (**Figure 1**) (Breitbart et al., 2018; Gregory et al., 2020). Following sequencing of the VLPs, the lifestyle assignment of assembled viral contigs is based upon the presence of essential lysogeny genes, including integrase, lysogenic recombination, prophage proteins, and transposases (Shkoporov et al., 2019; Van Espen et al., 2021). However, the detection of these genes may be dubious due to genome fragmentation and the lack of extensive viral sequence databases (Gregory et al., 2020). Moreover, it is typical that the vast number of VLP-associated contigs cannot be assigned at a taxonomic level (Shkoporov et al., 2019). To overcome this, VLPs-associated contigs can be clustered into approximately genus- or subfamily-level groups known as viral clusters (VC) (Shkoporov et al., 2019). A limitation of this approach is that it can group mixed populations of virulent and temperate phages (Campbell et al., 2020), which further confuses the abundance and detection of temperate phages in the gut (**Figure 1**). Hence, the characterization of temperate phages in wide-scale studies is likely undermined.

Here, we review the role of phages in the human gut, presenting the insights on temperate phages based on both culture-dependent and -independent methods. First, we give an overview of the wealth of knowledge on the gut viral community. We next explore the potential roles of temperate phages in the gut. Finally, we highlight the relevance of integrating experimental and computational techniques to improve our understanding of temperate phages and their potential use in medical interventions.

## The gut viral community from a metagenomic perspective

### Where are they living? And who are they?

Viral populations inhabit the totality of the mammalian gastrointestinal tract (Shkoporov et al., 2022a). A recent study provided one of the highest-resolution demarcation of the mammalian virome to-date across two species; the domestic pig and rhesus macaque (Shkoporov et al., 2022a). These authors found a strong partition between the virome inhabiting the large intestine and other gastrointestinal locales, with the large intestine virome being more abundant, diverse, and shared between the caecum and colon, compared with the lower abundance and relatively confined viromes of the small intestine and stomach (Shkoporov et al., 2022a). The large intestine luminal content of both domestic pigs and macaques mainly harbored tailed bacteriophages (Caudoviricetes), including crAss-like phages and ssDNA Microviridae, with a lower fraction of eukaryotic viruses (families Circoviridae, Astroviridae, Caliciviridae and Parvoviridae) (Shkoporov et al., 2022a). Furthermore, total viral loads in the large intestine mucosa samples were three orders of magnitude lower than matched luminal samples (Shkoporov et al., 2022a).

Similar to the enteric viral composition in rhesus macaques and domestic pigs, the adult human gut virome is dominated by phages, with more than 97% of the assigned contigs identified as phage (Gregory et al., 2020; Garmaeva et al., 2021; Van Espen et al., 2021). Gut phage populations overwhelmingly consist of Caudoviricetes -including crAss-like phages- and Microviridae phages (Shkoporov et al., 2019; Gregory et al., 2020; Zuo et al., 2020; Garmaeva et al., 2021; Adiliaghdam et al., 2022; Gulyaeva et al., 2022). Moreover, phages from the Inoviridae family along with novel candidate families Flandersviridae and Quimbyviridae, which infect *Bacteroides*, *Parabacteroides*, and *Prevotella*, are commonly found in the gut but at lower abundances (Shkoporov et al., 2019; Gregory et al., 2020; Zuo et al., 2020; Benler et al., 2021; Garmaeva et al., 2021; Adiliaghdam et al., 2022). As such, the human gut virome is highly individual and temporally-stable, where a predominant yet small fraction of viruses that persist over time (Shkoporov et al., 2019; Dahlman et al., 2021; Van Espen et al., 2021). Shkoporov et al. named this fraction as the persistent personal virome, where ~2% of the total viral contigs recruited a median of ~90% of the VLPs-reads per sample and were present in at least six out of 12 monthly samples collected from ten adults (Shkoporov et al., 2019). Similarly, Van Espen et al. showed that the ten most abundant viral contigs represented a median of ~80% of the total reads per sample from 91 individuals, including 46 children and 45 adults (Van Espen et al., 2021). These results indicate that the individual gut viral signature is captured by relatively few yet abundant viral contigs.

### When does the individual viral signature appear? And what is its relation with disease states?

The individual uniqueness of the virome begins immediately after birth and may have broad implications on the development of a healthy gut ecosystem. New-born babies have a high interpersonal variation in their virome (Maqsood et al., 2019). Indeed, the interpersonal variation seen in the virome among 1-4 day-old new-born babies is significantly higher compared to the variation among their mothers (Maqsood et al., 2019). This high interpersonal variation is further found in children, adolescents, adults, and even in 25-week old preterm infants (Gregory et al., 2020; Liang and Bushman, 2021; Van Espen et al., 2021; Beller et al., 2022; Kaelin et al., 2022). Preterm infants are particularly susceptible to develop necrotizing enterocolitis (NEC), a disease with a case mortality ranging between 22 to 38% (Kaelin et al., 2022). Surprisingly, it was found that ten days prior to the onset of NEC the virome beta diversity among preterm infants converged with the enrichment of specific viruses (Kaelin et al., 2022). This suggests that the collapse of the highly individualised gut virome followed by the expansion of specific viruses could be a biomarker of NEC disease states.

Phages can encode auxiliary metabolic genes (AMGs), and through this influence their bacterial host’s metabolic capacities and ecology, which may have implications in health and disease states (Breitbart et al., 2018; Mangalea et al., 2021). In particular, viromes of individuals at-risk for rheumatoid arthritis (RA) carry fewer phage-encoded AMGs than healthy controls (Mangalea et al., 2021). Notably, in individuals at-risk for RA, AMGs involved in the production of bacterial cell membrane polysaccharides and biofilm formation were less abundant compared to healthy controls, while AMGs involved in lipopolysaccharide and peptidoglycan biosynthesis were more abundant (Mangalea et al., 2021). This indicates that phages can drive bacterial surface modifications through AMGs, and potentially influence bacterial fitness (Mangalea et al., 2021), subsequent phage infectivity (Shkoporov et al., 2022b), and the stimulation of the immune system within the gut (Mangalea et al., 2021). Additionally, it was found that the virome of centenarians carry AMGs related to sulphur metabolic pathway, suggesting a higher microbial output of hydrogen sulphide that has been reported to promote colonization resistance and protection against aerobic pathogens (Johansen et al., 2023).

### Studying gut temperate phages

The investigation of VLPs-enriched metagenomes has substantially contributed to our understanding of phages in the gut, however, these studies are inherently limited to virulent phages and induced temperate phages (Breitbart et al., 2018; Gregory et al., 2020). Comparatively, bulk metagenomes screen the entire microbial community and with sufficient depth are capable of sequencing both extracellular phage virions and the integrate prophages residing within bacterial host chromosomes (**Figure 1**). Thus, bulk and VLPs-enriched metagenomes capture different viral populations and evidence of this discrepancies have been observed (Gregory et al., 2020). By using matched bulk and VLPs-enriched metagenomes from the Shokoporov et al. cohort (Shkoporov et al., 2019), Gregory et al. detected a higher fraction of virus in the bulk metagenomes and observed that only 8.5% of the viral populations were recovered for both bulk and VLPs-enriched metagenomes (Gregory et al., 2020), highlighting that VLPs-enriched metagenomes only capture the induced temperate phages, herein termed extracellular temperate phages, and suggesting that a high proportion of temperate phages reside as prophages in their host (Gregory et al., 2020). Therefore, a coupled approach of bulk and VLP metagenomes might be the most suitable option to survey the whole gut community of temperate phages. However, the ease of extracting and processing bulk metagenomes, compared with VLP metagenomes, along with their enhanced capacity for viral recovery render them an attractive choice for the study of temperate phages and their respective infection stages.

By employing bioinformatic tools, it becomes feasible to examine both intra- and extra-cellular temperate phages within bulk metagenomes. Various packages, such as VirSorter (Roux et al., 2015), VIBRANT (Kieft et al., 2020), PHASTER (Zhou et al., 2011; Arndt et al., 2016), Prophinder (Lima-Mendez et al., 2008), ProphET (Reis-Cunha et al., 2019), PhySpy (Akhter et al., 2012), and Phigaro (Starikova et al., 2020), can be utilized to predict temperate phages. Subsequently, the lifestyle of the temperate phage can be estimated using PropagAtE (Kieft and Anantharaman, 2022) or PIE (Miller-Ensminger et al., 2023), which employ statistical analyses of prophage-to-host read coverage ratios, with a higher ratio being indicative of extracellular temperate phages. However, these packages have inherent limitations, including failure to identify novel phages and identification of false positives arising from cryptic prophages. Despite these limitations, the study of temperate phages from bulk metagenomes has the potential to provide insights into their composition and life cycles within the gut.

To move beyond predictions of temperate phages and begin to characterise and understand phage specificity (*i.e.*, what bacteria do these phages infect)? and their effect on the bacterial and mammalian host, it is essential to integrate wet lab techniques with sequencing approaches (Fitzgerald et al., 2021; Shamash and Maurice, 2022). Various bioinformatic tools, such as PHERI, HostPhinder, Host Taxon Predicter, WIsH, and CHERRY, leverage sequence similarity, CRISPR spacer matches, and deep learning models to predict phage-bacteria interactions (Shang and Sun, 2022; Ostenfeld et al., 2023). However, their effectiveness is limited to well-characterized phage-host pairs and host species, with false positive predictions remaining difficult to validate (Ostenfeld et al., 2023). To overcome these limitation, a range of experimental approaches, including traditional spot, plaque, and liquid assays, viral tagging, microfluidic PCR, phageFISH, single cell sequencing, and Hi-C sequencing can be employed to validate prophage predictions and begin to build experimental systems for further characterisation (Edwards et al., 2016). It is important to note that most of these methods, along with phenotypic and gene function characterization, are heavily dependent on culture-based techniques (Edwards et al., 2016; Ostenfeld et al., 2023). To this end, recent studies have begun to isolate, culture, and sequence gut bacterial isolates from faecal samples worldwide (Stokholm et al., 2016; Forster et al., 2019; Hryckowian et al., 2020; Camarillo-Guerrero et al., 2021). These studies not only provide the protocols to isolate novel gut bacteria but also provide novel temperate phages and their bacterial hosts as *in vitro* model systems, enabling insights into gut phage biology.

## Temperate phages insights from culture-independent and culture-dependent techniques

### The bloom of temperate phages after birth

During the first years of life, there is a striking increase in the abundance and richness of VLPs, the majority of which appear to be temperate (Liang et al., 2020; Beller et al., 2022; Shamash and Maurice, 2022). In the first ~four days postpartum, VLPs are not detected in the majority of meconium samples (Liang et al., 2020). However, in the following month of life, detectable VLPs expand in the gut reaching concentration of 10^9^ VLPs per gram of stool remaining at this abundance through to adulthood (Liang et al., 2020). In a Belgian study of eight infants, it was found that 76% of the sequenced VLPs were predicted to potentially have a temperate lifestyle (Beller et al., 2022). Notably, another study found that one to four day old babies share ~60% of their bacterial populations with their mothers’ while sharing just ~15% of their gut viruses (Maqsood et al., 2019). Thus, the establishment of the gut virome in the first years of life appears to be driven by an early bloom of extracellular temperate phages originating from pioneering maternal bacteria colonising the gut (Maqsood et al., 2019; Liang and Bushman, 2021; Taboada et al., 2021).

In a small collection of bacterial strains isolated from new-born babies, spontaneous induction of VLPs was observed in ~30% of isolates (Liang et al., 2020). Using the same bacterial strains, the number of induced strains was increased to 80% following the addition of the gold standard induction agent Mitomycin C (Liang et al., 2020). A similar study screened prophage induction across 900 *Escherichia coli* strains isolated from 648 faecal samples collected from 1-year-old infants and found >60% were capable of autoinducing prophages and forming plaques in two laboratory *E. coli* strains (Mathieu et al., 2020). These *in vitro* observations support the hypothesis that earlier gut phage populations originate from prophage-induction events of pioneer bacteria.

### Induction of temperate phages in the adulthood

During the transition from infancy to adulthood, a noticeable decline in extracellular temperate phages is observed in the gut (Beller et al., 2022). This decline reaches its peak during adulthood (18-60 years old), with a higher proportion of temperate phages undergoing lysogenic cycle, gradually shifting towards a preference for lytic cycles as individuals age (Johansen et al., 2023). In a cohort of Belgian infants, a decreasing abundance of the extracellular temperate phages was observed towards the end of the first year (Beller et al., 2022). Congruently, in a Danish population of 45 adults and 46 children, the alpha diversity of extracellular temperate phages was significantly decreased in adults compared to infants, with the relative abundance of extracellular temperate phages ranging from 0% to 68% (Shkoporov et al., 2019; Van Espen et al., 2021). This broad range in the relative abundance points out that external factors such as diet, age, use of medication, location, and disease states can affect either the initial number of prophages or the number of induction events per individual. Lastly, in a Japanese cohort, a higher ratio of temperate phages to bacteria was observed in centenarians compared to both elderly (<60 years old) and young adults (>18 years old), indicating a shift back to lytic cycle as individuals age (Johansen et al., 2023).

Prophages are differently induced by dietary compounds (Boling et al., 2020) and oral medications (Sutcliffe et al., 2021). While compounds such as fernet, Arabica coffee, and oregano reduced the number of VLP related to spontaneous inductions in isolated strains of *Bacteroides thetaiotaomicron*, *Enterococcus faecalis*, and *Staphylococcus. aureus*, other agents, such as toothpaste, were able to increase the induction of certain prophages (Boling et al., 2020). In fact, a decrease of integrase genes in VLPs-associated contigs was observed during the transition from a normal to high-fat diet in mice, suggesting an overall reduction of extracellular temperate phages was driven by diet (Schulfer et al., 2020). In the case of the gut lysogen *Lactobacillus reuteri*, dietary fructose or gut-derived short chain fatty acids drove the induction of its prophage via the Ack pathway in a RecA dependent manner (Oh et al., 2019). Moreover, oral medications divergently induced VLPs in three Bacteroidetes, three Firmicutes, and one Actinobacteria gut strains (Sutcliffe et al., 2021). These results suggest that both spontaneous and differential prophage induction occur in the gut and that these induction events are both strain- and compound-specific (Sutcliffe et al., 2021), and in consequence, prophage inductions may differ between individuals based on its diet and use of medication.

Further evidence suggests that induction of prophages are associated with IBD (Clooney et al., 2019). Temperate VCs differentially increased in patients with the two most common subtypes of IBD, Crohn’s disease (CD), and UC, relative to control subjects (Clooney et al., 2019). Moreover, temperate VCs recruit significantly more reads in CD patients than healthy controls (Clooney et al., 2019). Furthermore, 15 out 17 *Siphoviridae* contigs increased in IBD patients were classified as temperate phages of *Firmicutes* (Clooney et al., 2019). These results correspond with the reduced Firmicutes abundance in IBD and provides evidence that induction of specific temperate phages may directly alter their host abundance and association with IBD (Clooney et al., 2019).

### How do temperate phages interact with bacterial host and epithelial cells?

Integrated prophages can modulate the metabolism of their bacterial host and encode genes to induce epithelial cellular responses (Campbell et al., 2020; Brown et al., 2021). The prophage BV01 was found to alter the bile acid metabolism of its host, *Bacteroides vulgatus*. By integrating into the *tspO* promoter, phage BV01 disrupted the genes function and repress the bile acids deconjugation, suggesting a regulatory link between TspO and the hydrolysis of bile acids (Campbell et al., 2020). Bile acids are secreted by the mammalian cells at high concentration in the small intestine, facilitate fat absorption, and alterations on their metabolism are associated with obesity (Campbell et al., 2020; Li R. et al., 2021). Furthemore, it was shown that both bile acids and oxidative stress induce the production of Bxa in *Bacteroides* gut isolates (Brown et al., 2021). Bxa is a *Bacteroides-*encoded ADP-ribosyltransferase, which is known to be encoded by a prophage within select *B. stercoris* strains (Brown et al., 2021). The expression of Bxa in *B. stercoris* lysogens is co-activated with genes relevant to bacterial adhesion and secretion, and importantly, secreted Bxa induces the gut epithelial cells to secrete inosine, which further promotes *B. stercoris* growth and biofilm formation (Brown et al., 2021). Notably, phage BV01 was found to integrate into the genome of another bacterial host exclusively in a gnotobiotic mouse model (Campbell et al., 2020), highlighting the presence of unexplored tripartite biological factors necessary for successful infection that are not effectively replicated under *in vitro* conditions. This wet-lab characterization shows the co-dependent relationships between prophages, bacteria, and the human host.

## Conclusion

Bulk and VLPs-enriched metagenomes continue to be pivotal in our understanding of temperate phages within the gut. While we are beginning to uncover associations between temperate phages and gut health, it is important to recognise the inherent limitations when utilising these sequencing approaches, particularly the bias towards isolating either the intracellular or extracellular lifecycles of temperate phage. The next steps towards the understanding of these viruses must hybridize both culture-based techniques with sequencing approaches to unveil the biology and mechanistic insights of gut temperate phages, their ecology and associations with human health.

## Author contributions

LA-F: Conceptualization, investigation, writing, visualization - original draft. SD: Conceptualization, investigation, writing - review and editing. JB: Conceptualization, investigation, writing - review & editing, supervision, funding acquisition. All authors contributed to the article and approved the submitted version.
